# The impact of family endowment on adolescents’ physical activity behavior

**DOI:** 10.3389/fpubh.2025.1589515

**Published:** 2025-06-04

**Authors:** Shanshan Xie, Na Li, Wenxia Guo

**Affiliations:** ^1^China Women's University, Beijing, China; ^2^School of Physical Education, Shandong University of Science and Technology, Qingdao, China; ^3^College of Humanities and Law, Beijing University of Chemical Technology, Beijing, China

**Keywords:** family endowment, physical fitness behavior, family sports, adolescents’ physical fitness, social support

## Abstract

**Objective:**

The cultivation of adolescent lifelong development is significantly influenced by family education, yet current approaches to physical activity promotion reveal critical systemic deficiencies. Family sports education remains underprioritized, while school-based physical education programs demonstrate diminishing efficacy. This situation is exacerbated by the absence of collaborative frameworks integrating community sports resources, collectively contributing to suboptimal sports participation rates among Chinese youth. Given the critical role of family resources in enhancing adolescent physical fitness outcomes, this study aims to: (1) identify key family capital dimensions influencing youth sports engagement; (2) examine the practical requirements for family-oriented sports initiatives; and (3) propose evidence-based strategies to address the multifaceted physical fitness challenges confronting Chinese adolescents.

**Methods:**

A random cluster sampling method was used to select 9 cities from the 17 cities in Shandong Province as the survey objects, and the two-class and ordered multi-class Logistic regression analysis was used to analyze the influence of family endowment on adolescents’ physical fitness behavior.

**Results:**

(1) The results of binary logistic regression analysis showed that the mother’s higher education level was more conducive to the formation of adolescents’ physical fitness behavior; the health of fathers affects adolescents’ physical fitness behaviors; the annual family income of “11–20 Ten thousand” has a significant impact on whether to participate in physical fitness, other groups have no statistical difference; the number of relatives and friends except for the “1–3” group, other groups have significant difference; “3–5″ and “6 or more” in the quality of the relationship network are all influencing factors of adolescents’ physical fitness behavior; there was no statistical difference in the influence of family activity space and family debt level on adolescents’ physical fitness behavior. (2) The results of multivariate ordinal logistic regression analysis showed that the educational level of mothers promoted the physical fitness needs of adolescents, the increase of family activity space promoted the physical fitness needs of adolescents, and the improvement of family income level also contributed to the physical fitness of adolescents. The promoting factors of fitness behavior, and the number of relatives and friends and the quality of the relationship network affect the physical fitness behavior of adolescents, and the differences are statistically significant.

**Conclusion:**

Family endowment positively impacts adolescents’ physical fitness behaviors, with human, natural, economic, and social capital all influencing their sports participation. Establishing a family-focused sports environment and a robust social support system is essential. Moreover, special attention is needed for adolescents with low parental education levels in order to improve family fitness awareness and behaviors.

## 1 Introduction

The COVID-19 pandemic exacerbated existing concerns about the declining physical fitness of adolescents while underscoring the pivotal role of families in shaping health behaviors. During prolonged periods of “home epidemic prevention,” families became the primary facilitators of physical activity, revealing both their potential and shortcomings. Households were tasked with compensating for the suspension of school and community sports, yet systemic weaknesses in family-centric sports education-such as inconsistent parental involvement, limited access to home-based fitness resources, and a lack of structured guidance—became apparent. Notably, the Family Education Law of the People’s Republic of China (1) marked a paradigm shift by redefining family education as a societal responsibility rather than a private matter. This legal framework emphasizes the need for families to actively cultivate health literacy, including sports participation, positioning households as critical agents in addressing post-pandemic fitness gaps. However, while families are now recognized as central to adolescent well-being, their capacity to fulfill this role remains uneven, necessitating urgent institutional and theoretical support. Secondly, China’s exam-oriented education system has long marginalized physical education (PE), reducing it to a peripheral activity. A decade after the implementation of Central Document No. 7 (2007), which aimed to revitalize school sports, students still lack fundamental skills such as swimming, speed running, and ball games. Risk-averse policies have further diluted PE curricula into “three no” (no intensity, no confrontation, no collisions) and “seven no” (no sweating, no panting, etc.) classes, prioritizing safety and academic time over physical development. Compounding this issue, a 2020 Ministry of Education survey reported an 11.7% surge in student myopia rates within 6 months—a direct consequence of inadequate outdoor activity (2). Schools, constrained by curricular demands and liability concerns, struggle to provide the mandated daily hour of outdoor exercise, shifting the onus onto families to bridge this gap. Yet, without a cohesive collaboration between families, schools, and communities, systemic barriers persist, undermining adolescents’ holistic development. To address these challenges, this study introduces family endowment-a composite of familial resources, cultural capital, and intergenerational practices that shape health behaviors. Rooted in sociological theories such as Bourdieu’s cultural capital (1986) (3), family endowment encompasses economic means (e.g., access to sports facilities), parental health literacy, and value systems prioritizing physical activity. For instance, families with robust endowments may model active lifestyles, enroll children in extracurricular sports, or advocate for school-community partnerships. Conversely, economically or educationally disadvantaged households often lack such leverage, perpetuating inequities (4). Literature underscores that family endowment significantly predicts adolescent sports participation, particularly when institutional systems falter (5). In the post-pandemic context, leveraging this framework can inform policies that empower families through resource allocation, parental education programs, and cross-sectoral collaboration, ultimately fostering sustainable co-education mechanisms for youth fitness.

Physical activity serves as a protective behavior for adolescents, shaped by interactions between external socio-environmental factors and internal psychological-physiological mechanisms. Psychological determinants, such as sports-related attitudes and emotional responses during moderate-intensity exercise, significantly predict sustained engagement in physical activity. Externally, the family functions as the primary agent of socialization during early developmental stages, exerting enduring influence on sports behavior through parental encouragement, role modeling, and shared activities (6). As a microenvironment complementary to formal education, the family endowment fosters physical skill acquisition and integrates sports into daily life, thereby reinforcing health behaviors. This environment encompasses material resources, social status, interpersonal dynamics, and behavioral patterns, with parental education levels and socioeconomic status frequently identified as critical predictors of adolescent physical activity (7). Given its irreplaceable role in shaping health trajectories, family-centric interventions should be prioritized within holistic strategies to promote adolescent sports engagement.

Family endowment refers to the collective resources and capabilities inherent to a household, structured across four interrelated dimensions: (1) human capital (e.g., parental education, health literacy), (2) natural capital (e.g., access to recreational spaces, sports facilities), (3) social capital (e.g., familial networks, community ties), and (4) economic capital (e.g., income, material assets) (8). As the primary agent of early socialization, families shape adolescents’ physical activity behaviors through guidance, intergenerational modeling, and resource allocation (9). Parental health consciousness and the household’s capacity to foster exercise-friendly environments significantly influence children’s sports cognition, participation levels, and long-term behavioral tendencies (10). Bourdieu’s capital theory (1986) provides a robust framework for analyzing how family resources shape adolescent physical activity behaviors (PA). Bourdieu’s conceptualization of capital-economic (material assets), cultural (knowledge, values), and social (networks, relationships)-aligns closely with our operationalization of family endowment. Specifically, economic capital reflects a family’s financial capacity to invest in sports equipment, facilities, and structured PA opportunities. Cultural capital manifests as parental health literacy, attitudes toward exercise, and the intergenerational transmission of active lifestyles, which collectively normalize PA as a valued family practice. Meanwhile, social capital encompasses familial and community networks that facilitate access to sports clubs, mentors, or peer groups, enabling adolescents to sustain engagement through social reinforcement. Building on this framework, we demonstrate how the unequal distribution of these resources perpetuates disparities in PA participation. By grounding family endowment in Bourdieu’s theory, this study elucidates the structural mechanisms underlying adolescent PA inequities, offering actionable insights for mitigating resource-driven health divides.

Physical activity is widely recognized as a protective behavior for adolescents, mitigating risks of social anxiety, sleep disorders, and sedentary lifestyles—a finding corroborated globally, including in studies of young e-sports athletes (11). While family economic and cultural capital significantly shape physical activity patterns, disparities in adolescent sports participation often stem from unequal family endowments. Higher socioeconomic status enables greater parental investment in sports equipment, structured exercise time, and role modeling, which directly enhance adolescents’ engagement (12). Parental education levels further amplify this effect, as educated caregivers are more likely to prioritize physical health literacy and foster supportive environments (13). However, existing research predominantly examines isolated family factors (e.g., income, education) rather than adopting a holistic view of family endowment-a multidimensional construct encompassing human, social, natural, and economic capital. This gap limits actionable insights for addressing systemic inequities in youth fitness, particularly in contexts like China, where rising myopia and academic pressures exacerbate physical inactivity.

Building upon existing research, this study systematically examines the influence of family endowments on adolescents’ physical fitness behaviors through field investigations and synthesis of domestic/international literature. Utilizing logistic regression modeling, we identify key familial determinants across four capital dimensions (human, natural, social, and economic) and elucidate implementation barriers in family-based health promotion. Our contributions advance current scholarship through two principal innovations: (1) Theoretical expansion: as the first investigation to holistically analyze adolescent exercise behaviors through the lens of multidimensional family capital, this research establishes an original conceptual framework linking household resource allocation to youth physical activity patterns. This approach not only clarifies mechanisms of familial influence on health behaviors but also enriches theoretical foundations in family sports science and adolescent health interventions (2). Methodological rigor: implementing a psychometrically validated family endowment questionnaire with a larger-scale, regionally representative sample (*N* = 1,025), we demonstrate enhanced methodological robustness compared to previous localized studies. The application of multivariate logistic regression provides empirical evidence quantifying relative effect sizes of distinct capital types, offering policymakers actionable insights for targeted family support programs.

## 2 Data sources and study methods

### 2.1 Data source and sample description

The study mainly used Shandong Province of China as the research area. Based on the research variables, regional economic development levels, and geographical distribution, a random cluster sampling method was employed to select 9 cities from the 17 prefecture-level cities in Shandong Province as the survey area, ensuring proportional representation across different economic development tiers, namely, including Taian City, Jining City, Qingdao City, Binzhou City, Jinan City, Liaocheng City, Rizhao City, Linyi City, and Weifang City. The inclusion criteria required participants to: (1) be aged 12–14 years, (2) currently enrolled in grades 7–9 (junior high school), and (3) have resided in the designated survey areas throughout the study period.

Data collection was implemented through parent/guardian-administered questionnaires to ensure validity and compliance. This study was conducted in accordance with the ethical principles of the Declaration of Helsinki and received formal approval from the Institutional Review Board (IRB) of China Women’s University. Written informed consent was obtained from all participating parents/legal guardians prior to data collection. The consent form explicitly outlined: (1) The research objectives and methodology; (2) Voluntary participation and the right to withdraw at any stage; (3) Data anonymization procedures to ensure confidentiality; (4) Potential risks/benefits of participation.

The data includes the basic economic situation of teenagers’ families, social capital, production, and income. Initially, a pilot survey was conducted to examine response patterns in the adolescent population. The validity of the collected data was systematically evaluated. Subsequent test–retest reliability assessments demonstrated the questionnaires’ satisfactory internal consistency (Cronbach’s *α* = 0.85). Normality assumptions were verified through Kolmogorov–Smirnov tests (*p* > 0.05) with computed skewness (−0.96 to 0.92) and kurtosis (−1.25 to 1.01) values falling within acceptable ranges (±2), confirming parametric analysis suitability. A stratified random sampling method was then implemented to ensure population representativeness, distributing and collecting questionnaires via Wenjuanxing (an online survey platform). A total of 1,079 questionnaires were obtained. Questionnaires that were incomplete, had large unanswered sections, or contained outlier values were excluded from the analysis, resulting in 1,025 valid responses (effective response rate = 95%). The cohort consisted of 527 female (51.4%) and 498 male (48.6%) participants.

### 2.2 Variable selection and analysis method

#### 2.2.1 Variable selection

The research takes teenagers’ physical fitness behavior as the dependent variable. Teenagers’ physical fitness behavior is defined as purposeful engagement in structured physical activities (e.g., exercise, sports, or movement-based practices) designed to enhance physical and mental well-being. It serves as an observable indicator of adolescents’ participation in physical activity and plays a pivotal role in promoting sustained sports involvement and mitigating health risks associated with sedentary lifestyles, such as declining physical fitness. As a measurable indicator of sports engagement levels, this behavioral pattern significantly influences fitness improvement and sustained athletic involvement. The present research focuses on analyzing the prevalence and frequency patterns of these fitness behaviors within adolescent populations (14). Family endowment is the expansion of family members’ personal abilities and resources, which is the sharing of the abilities and resources possessed by the family (15). Different scholars have provided interpretations of this concept, mostly from the aspects of human capital, natural capital, economic capital, social capital, etc. This study defines family endowment as the resources that can be shared by families and their members, and analyzes teenagers’ physical fitness behavior from four aspects. Human capital refers to the accumulation of knowledge and skills acquired by families through investment in education, health, and training (16). Therefore, the study selects the average education level and health status of parents to reflect the quality and quantity of family human capital. The higher the education level of parents, the more comprehensive their understanding of the importance of health and physical fitness, the higher the requirements for children’s physical fitness, and the greater the degree of support may be. The health status of family members may have a positive impact on teenagers’ physical fitness behavior. In a broad sense, natural resources include the means of production and living derived from nature, including renewable resources such as trees and livestock, as well as non-renewable resources such as crude oil. This study refers to the resources owned by families that can be used for teenagers’ physical fitness, mainly the total area of activity space owned by families, which may have a low relationship with urban teenagers’ physical fitness behavior, so the expected impact cannot be estimated. Economic capital mainly refers to the economic status and economic strength of the family. The study selected the level of family income and debt to measure it. It is believed that the high level of family income will lead to less pressure on living expenditure, which will enhance the support for teenagers’ sports and fitness, and may have a positive effect on it. Family debt will affect the normal living expenditure of the family, so it may reduce the investment and companionship for teenagers’ sports and fitness, and the expected effect is negative. Social capital mainly refers to the status of families in their social networks and the social resources available to them (17). The number of relatives and friends is selected as the size of the social network. The size of the social network may affect the spread of fitness concepts and correct health concepts, but the quality of the social network is also an important factor of social capital. Different occupations provide different information, and the social resources obtained will be different. Therefore, defining high-quality social relationships as the nature of work among relatives and friends, the more people who have high-quality social relationships among relatives and friends, the more fitness and health information they get, the more likely they will positively influence the physical fitness and health behaviors of adolescents (Table 1).

**Table 1:** Variable description and descriptive statistics.

Variable type	Variable name	Symbol	Variable assignment
Dependent variable	Physical fitness behavior	*y*	No demand = 1, In demand = 2
	Participation degree	*y*_1_	One time = 1, 2–3 Times = 2, More than 3 times = 3
Independent variable	Father’s education level	X_1_	Primary school and below = 1, Junior middle school = 2, High school or technical secondary school = 3, College degree or above = 4
	Mother’s education level	X_2_	Primary school and below = 1, Junior middle school = 2, High school or technical secondary school = 3, College degree or above = 4
	Father’s health	X_3_	Very good health = 1, Healthy = 2, Subhealthy = 3, Ill health = 4
	Mother’s health	X_4_	Very good health = 1, Healthy = 2, Subhealthy = 3, Ill health = 4
	Family activity space	X_5_	Total area of family owned activity space (*m*_2_)
	Family income	X_6_	Annual family income level(Ten thousand yuan) 1 = ≤1, 2 = 1–10, 3 = 11–20, 4 = 21–30, 5= > 30
	Debt level	X_7_	Liabilities = 1, No liabilities = 0
	Number of relatives and friends	X_8_	Number of family relatives and friends
	Relationship network quality	X_9_	Number of relatives and friends engaged in education, government, institutions and physical education training (1 = no, 2 = 1–2, 3 = 3–5,4 = 6 or more)
Control variable	Gender	Z	1 = Female, 2 = Male

#### 2.2.2 Analytic procedure

Spss24.0 was used to make descriptive statistics on the data, the X^2^ test was used to analyze the categorical variables, binary logistic regression analysis was used to analyze the influencing factors of teenagers’ physical fitness behavior, and ordered multiclass logistic regression was used to analyze the degree of teenagers’ participation in physical fitness activities.

Prior to regression modeling, variance inflation factors (VIFs) were computed for all predictor variables to evaluate potential multicollinearity. Following established thresholds (VIF < 5.0), all values fell below 3.0, indicating no substantive multicollinearity concerns. We simultaneously assessed model fit indices for the ordinal logistic regression analyses, including Nagelkerke’s pseudo R^2^ (0.12) and McFadden’s pseudo R^2^ (0.18), to comprehensively characterize model performance and explained variance.

1. The dependent variable is teenagers’ physical fitness behavior, which is a qualitative binary variable. Therefore, a binary logistic regression model was used for analysis. To observe the influence of different dimensions of family endowments on teenagers’ physical fitness behavior, while controlling for variables such as human capital, social capital, natural capital, and economic capital, models were constructed (Equation 1).

(1) $$\ln\;y=\ln(\frac{p}{1-p})=\beta_{0}+\sum_{i}\beta_{i}x_{i}+\mu$$

Where *y* represents the physical fitness activities of adolescents, *p* represents the probability of adolescents engaging in physical fitness activities, and 1−*p* represents the probability of adolescents not engaging in physical fitness activities. *x_i_* denotes the ith explanatory variable, *β_i_* represents the constant term, *β_i_* represents the regression coefficient for each explanatory variable, μrepresents the random error term, and i = 1, 2, …, 9. Calculate the correlation between the influencing factors and the physical fitness activities of adolescents, and conduct a significance test.

1. The dependent variable of adolescent physical fitness behavior participation degree was assigned values 1,2,3 respectively, which was analyzed by orderly multi-classification Logistic regression (Equations 2, 3). The formula is as follows:

(2) $$\log\mathrm{it}\{p(y\rangle i/x)\}=-B_{i}+B_{1}X_{1}+B_{2}X_{2}+\ldots \ldots \ldots +B_{n}X_{n}$$

(3) $$P\;(y\leq i/x)=\frac{1}{1+\exp[-B_{i}+B_{1}X_{1}+B_{2}X_{2}+\ldots \ldots \ldots +B_{n}X_{n}]}$$

$$\begin{array}{l} P\;(y=i/x)=P\;(y\leq i/x)-(y\leq i-1/x)\\ =\frac{1}{1+\exp[-B_{i}+B_{1}X_{1}+B_{2}X_{2}+\ldots \ldots \ldots +B_{n}X_{n}]}\\ -\frac{1}{1+\exp[-B_{i-1}+B_{1}X_{1}+B_{2}X_{2}+\ldots \ldots \ldots +B_{n}X_{n}]} \end{array}$$

In formula, *y* is the participation degree of adolescent physical fitness behavior of the dependent variable, X_i_ (i = 1,2,3… n) represents the independent variable, indicating the family endowment factors affecting the participation degree, B is the regression coefficient, which is used to explain the influence degree and direction of each factor of the independent variable on the dependent variable.

Binary logistic regression was applied to dichotomous outcomes (e.g., achievement/non-achievement of physical activity thresholds), where the probability of event occurrence was modeled. Ordered multiclass logistic regression was chosen for graded outcomes to preserve the inherent ordering of categories while assessing cumulative probabilities across thresholds. This approach optimally balanced interpretability, statistical power, and alignment with our hypothesis-testing framework.

## 3 Results

### 3.1 Single factor analysis of teenagers’ physical fitness behavior

A total of 1,025 surveys were included in the analysis. The descriptive statistical results are presented in Table 2. In the past month, 48.8% (500/1025) of teenagers participated in physical fitness activities. The X2 test or rank sum test was employed to compare various indicators related to teenagers’ family endowments. The results of the single-factor analysis indicated that, among indicators X1-X9, except for X1 (father’s education level) and X4 (mother’s health status), all other indicators were significantly associated with teenagers’ participation in physical fitness activities (*p* < 0.05). These findings were statistically significant (Table 3).

**Table 2:** Statistical description and simple correlation analysis (*n* = 1,025).

Dimension	Variable		Total [n(%)]	Partake [n(%)]	nonparticipation [n(%)]	*X*	*p*-value
Human capital	X_1_ Father’s education level	Primary school and below	150 (14.6)	82 (54.7)	68 (45.3)	12.13	0.070
		Junior middle school	621 (60.6)	302 (48.6)	319 (51.4)		
		High school or technical secondary school	189 (18.4)	78 (41.3)	111 (58.7)		
		College degree or above	92 (9)	68 (73.9)	24 (26.1)		
	X_2_ Mother’s education level	Primary school and below	166 (16.2)	62 (37.3)	104 (62.7)	51.686	0.000
		Junior middle school	664 (64.8)	312 (47)	352 (53)		
		High school or technical secondary school	115 (11.2)	58 (50.4)	57 (49.6)		
		College degree or above	80 (7.8)	68 (85)	12 (15)		
	X_3_ Father’s health	Very good health	307 (30)	270 (87.9)	37 (12.1)	12.342	0.002
		Healthy	583 (57)	501 (85.9)	82 (14.1)		
		Subhealthy	98 (9.6)	23 (23.5)	75 (76.5)		
		Ill health	37 (3.6)	10 (27)	27 (73)		
	X_4_ Mother’s health	Very good healthy	797 (77.8)	380 (47.7)	416 (52.3)	8.097	0.088
		health	183 (17.9)	101 (55.2)	82 (44.8)		
		Subhealthy	28 (2.7)	9 (32.1)	19 (67.9)		
		Ill health	17 (1.7)	10 (58.8)	7 (41.2)		
Natural capital	X_5_ Family activity space (sq.m.)	No active space	68 (6.6)	31 (45.6)	37 (54.4)	12.868	0.025
		≤10	110 (10.7)	54 (49.1)	56 (50.9)		
		10–20	255 (24.9)	117 (45.9)	138 (54.1)		
		>20	412 (40.2)	226 (54.9)	186 (45.1)		
Economic capital	X_6_ Family income (ten thousand yuan)	≤1	270 (26.3)	116 (43)	154 (57)	11.511	0.030
		1–10	639 (62.3)	315 (49.3)	324 (50.7)		
		11–20	86 (8.4)	52 (60.5)	34 (39.5)		
		21–30	17 (1.7)	9 (52.9)	8 (47.1)		
		31–40	13 (1.3)	8 (61.6)	5 (38.4)		
	X_7_ Debt level	Yes	308 (30)	151 (49)	157 (51)	12.038	0.041
		No	717 (70)	349 (48.7)	368 (51.3)		
Social capital	X_8_ Number of relatives and friends (person)	No	24 (2.3)	10 (41.7)	14 (58.3)	21.948	0.001
		1–3	13 (1.3)	5 (38.5)	8 (61.5)		
		4–5	278 (27.1)	113 (40.6)	165 (59.4)		
		6–10	210 (20.5)	102 (48.6)	108 (51.4)		
		>10	233 (22.7)	142 (60.9)	91 (39.1)		
	X_9_ Relationship network quality (person)	No	638 (62.2)	251 (39.3)	387 (60.7)	64.287	0.000
		1–2	276 (26.9)	171 (62)	105 (38)		
		3–5	103 (10.0)	72 (70)	31 (30)		
		>6	8 (0.8)	6 (75)	2 (25)		
Control Variable	Female		527	51.4	——	——	——
	Male		498	48.6	——		

**Table 3:** Logistic regression analysis.

Dimension	Metric	B	Wald	*p*-value	OR
Human capital	Mother’s education level (Primary school and below)		15.985	0.003	
	Junior middle school	2.028	15.991	0.000	7.60***
	High school or technical secondary school	1.763	13.301	0.000	5.83***
	College degree or above	1.706	9.784	0.002	5.51**
	Father’s health (Ill health)		4.257	0.027	1.00
	Very good health	1.489	3.973	0.046	4.43*
	Healthy	1.492	3.759	0.041	4.45*
	Subhealthy	−1.799	3.491	0.066	0.17
Natural capital	Family activity space (sq.m.) (No active space)		7.963	0.158	1.00
	<10	−0.163	0.105	0.746	0.85
	10–20	−0.648	3.017	0.082	0.52
	>20	0.048	0.029	0.865	1.05
	Other	−0.487	2.531	0.112	0.61
Economic capital	Family income (ten thousand yuan) (≤1)		3.662	0.044	1.00
	1–10	0.779	0.463	0.496	2.18
	11–20	0.98	3.053	0.005	2.66**
	21–30	1.058	1.261	0.262	2.88
	>30	1.166	1.362	0.243	3.21
	Liabilities level (Yes)		0.404	0.817	1.00
	No	−0.418	0.229	0.632	0.66
Social capital	The number of relatives and friends (person) (No)		2.958	0.045	1.00
	1–3	0.801	0.502	0.479	2.23
	4–5	0.868	3.327	0.034	2.38*
	6–10	0.142	3.256	0.013	1.15*
	>10	0.073	3.973	0.008	1.08**
	Relationship network quality(person)(No)		7.358	0.018	1.00
	1–2	2.251	4.21	0.137	9.50
	3–5	0.615	6.45	0.043	1.85*
	>6	0.995	7.168	0.028	2.70*
	Constant	6.169	15.306	0.000	477.71

*p < 0.05, **p < 0.005, Liabilities level: Yes = Have a debt, No = No debt.

### 3.2 Binary logistic analysis on the influence of teenagers’ physical fitness behavior

Univariate analysis has identified the following key factors influencing adolescents participation in sports and fitness activities: mother’s education level, father’s health, family activity space, family income level, family debt level, the number of relatives and friends, and relationship network quality. Consequently, Taking the participation of teenagers in physical fitness activities in the past month as the dependent variable and the indicators of family endowment as the independent variables, a binary logistic analysis was conducted. The results showed that the Hosmer-Lemeshow test significance level was 0.883, indicating a good fit between the data and the model, with a prediction accuracy of 85.7%. Among the indicators of mother’s education level, compared to the primary school and below group, the junior high school group (OR = 7.60, *p* < 0.001), the high school or technical secondary school group (OR = 5.83, p < 0.001), and the college and above group (OR = 5.51, *p* < 0.005) showed significant differences, indicating that an increase in mother’s education level is associated with greater participation in physical fitness among teenagers. Among the indicators of father’s health status, compared to the unhealthy group, the very good health group (OR = 4.43, *p* < 0.05) and the healthy group (OR = 4.45, *p* < 0.05) showed significant differences, while the sub-healthy group showed no significant differences, indicating that father’s health status is an influencing factor in promoting teenagers’ participation in sports. Among the indicators of family income level, the group with an annual family income of 110,000–200,000 yuan (OR = 2.66, *p* < 0.01) had a significant impact on whether to participate in physical fitness, while no significant differences were observed in other groups. Significant differences were observed in the number of relatives and friends, except for those in the 1–3 group (OR = 2, *p* < 0.05; OR = 1.15, *p* < 0.05; OR = 1.08, *p* < 0.01). The relationship network quality of “3–5” (OR = 1.85, *p* < 0.05) and “more than 6” (OR = 2.70, *p* < 0.05) were identified as influencing factors for teenagers’ sports fitness behavior. No significant differences were observed in the influence of family activity space and family debt level on teenagers’ sports fitness behavior (Table 3).

### 3.3 Multivariate logistic analysis on the influence of adolescents’ participation in physical fitness behavior

After identifying the factors affecting teenagers’ physical fitness behavior, the study further analyzed the participation of people with physical fitness behavior needs using multi-factor ordinal logistic regression, in order to understand the influence of factors related to family resources on teenagers’ participation in sports activities.

**Figure 1:**
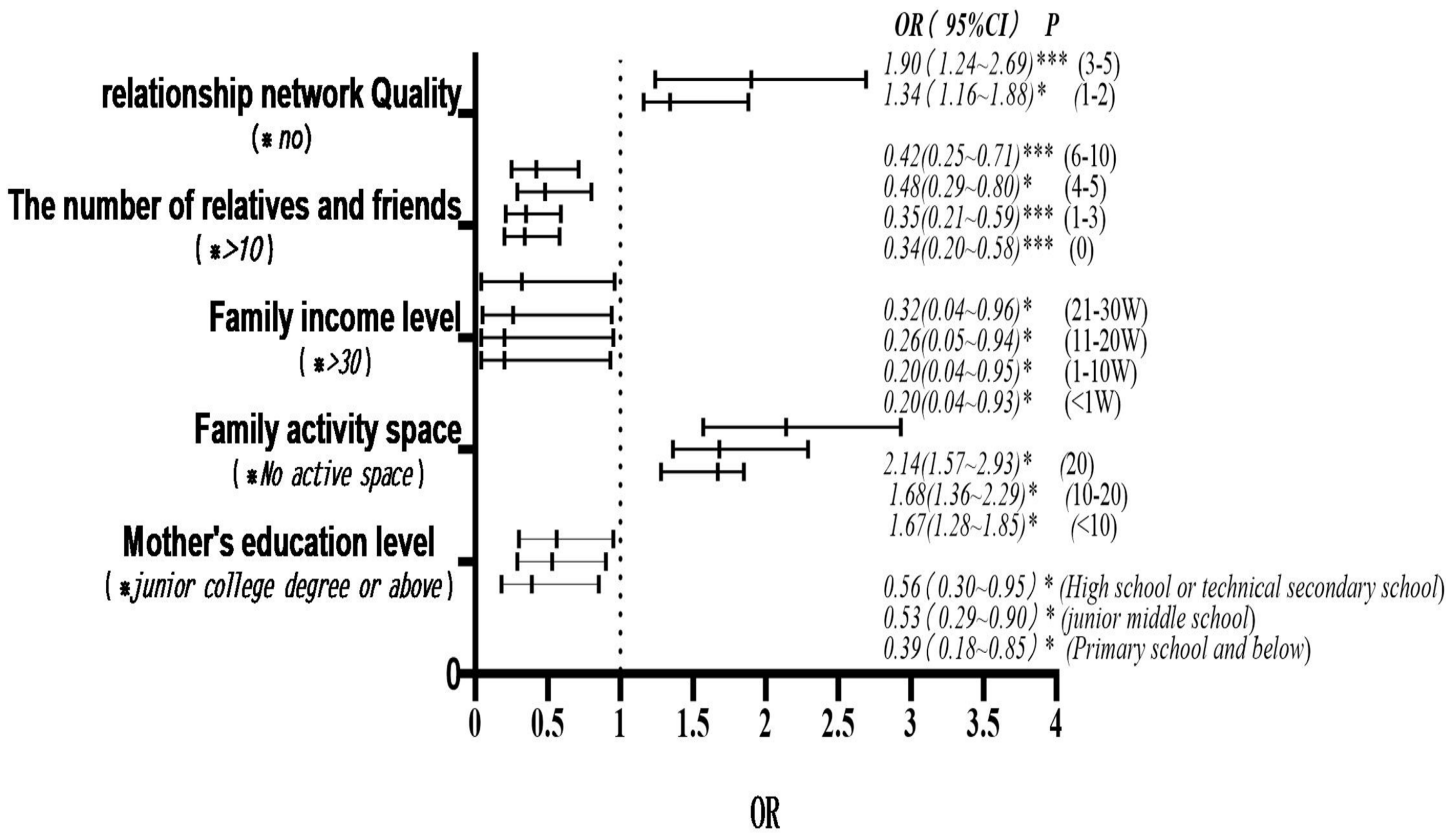
Results of the multivariate ordinal logistic regression analysis.

With fitness participation as the dependent variable, and sequentially included the mother’s education level, father’s health status, family activity space, debt level, number of relatives and friends, and quality of relationship network as independent variables, the impact of various family endowment indicators on adolescents’ participation in physical fitness activities was assessed using the X2 test. The results of the single-factor analysis indicated that, except for X3 (father’s health status) and X7 (debt level), all other indicators were significantly correlated with participation (*p* < 0.05), suggesting statistical significance. Based on the results of the independent variable analysis, a multi-factor ordinal logistic regression equation was constructed, incorporating the mother’s education level, family activity space, family income level, number of relatives and friends, and quality of relationship network (Table 4). The results indicated that *p* > 0.05, satisfying the parallel line test. An increase in the mother’s education level promotes the physical fitness needs of adolescents (OR = 0.39, 95%CI = 0.81–0.85, *p* < 0.05; OR = 0.53, 95%CI = 0.29–0.90, *p* < 0.05; OR = 0.56, 95%CI = 0.30–0.95, *p* < 0.05), showing statistical significance. An expansion in family activity space also boosts adolescents’ physical fitness needs (OR = 1.67, 95%CI = 1.28–1.85, *p* < 0.05; OR = 1.68, 95%CI = 1.36–2.29, *p* < 0.05; OR = 2.14, 95%CI = 1.57–2.93, *p* < 0.05), which is statistically significant. An increase in family income level also serves as a promoting factor for adolescents’ physical fitness (OR = 1.67, 95%CI = 1.28–1.85, *p* < 0.05; OR = 1.68, 95%CI = 1.36–2.29, *p* < 0.05; OR = 2.14, 95%CI = 1.57–2.93, *p* < 0.05), with the difference being statistically significant. An increase in the number of relatives and friends also enhances adolescents’ physical fitness needs, with the difference being statistically significant (OR = 0.34, 95%CI = 0.20–0.58, *p* < 0.001; OR = 0.35, 95%CI = 0.21–0.59, *p* < 0.001; OR = 0.48, 95%CI = 0.29–0.80, *p* < 0.001; OR = 0.42, 95%CI = 0.25–0.71, *p* < 0.001). Furthermore, the quality of the relationship network will enhance adolescents’ fitness needs (OR = 1.34, 95%CI = 1.16–1.88, *p* < 0.05; OR = 1.90, 95%CI = 1.24–2.69, *p* < 0.05; OR = 2.44, 95%CI = 1.32–3.15, *p* < 0.05), with the difference being statistically significant (Figure 1).

**Table 4:** Multi factor ordinal logistic regression analysis of adolescents’ participation in physical fitness behavior.

Dimension	Metric		B	Wald	*p*-value	OR	95%CI	
Requirement level		One time	−3.82	12.2	0.00	0.02	0.00	0.19
		2–3 time	−3.87	8.42	0.00	0.02	0.00	0.29
Human capital	Mother’s education level	Primary school and below	−0.93	5.70	0.02	0.39	0.18	0.85
		Junior middle school	−0.64	4.56	0.03	0.53	0.29	0.90
		High school or technical secondary school	−0.58	4.40	0.04	0.56	0.30	0.95
		College degree or above	0	–	–	–	–	–
Natural capital	Family activity space (sq.m.)	No active space	0	–	–	–	–	–
		<10	0.51	6.17	0.01	1.67	1.28	1.85
		10–20	0.52	1.72	0.02	1.68	1.36	2.29
		>20	0.76	3.43	0.04	2.14	1.57	2.93
Economic capital	Family income (ten thousand yuan)	≤1	−1.62	4.24	0.04	0.20	0.04	0.93
		1–10	−1.61	4.12	0.04	0.20	0.04	0.95
		11–20	−1.36	4.03	0.04	0.26	0.05	0.94
		21–30	−1.89	6.63	0.01	0.32	0.04	0.96
		>30	0	–	–	–	–	–
Social capital	The number of relatives and friends (person)	No	−1.07	15.72	0.00	0.34	0.20	0.58
		1–3	−1.04	15.34	0.00	0.35	0.21	0.59
		4–5	−0.73	8.04	0.01	0.48	0.29	0.80
		6–10	−0.86	10.49	0.00	0.42	0.25	0.71
		10	0	–	–	–	–	–
	Relationship network quality (person)	No	0	–	–	–	–	–
		1–2	0.29	5.99	0.01	1.34	1.16	1.88
		3–5	0.64	11.09	0.00	1.90	1.24	2.69
		>6	0.89	0.14	0.71	2.44	1.32	3.15

## 4 Discussion

### 4.1 The influence of family human capital endowment

Family human resources refer to the accumulation of knowledge and skills acquired in education, health, and training. Analyzing the quantity and quality of family human resources based on parents’ education and health levels, the results show that the mother’s education level and father’s health level (compared with the unhealthy group, parents who are healthy and “very good health” have a significant impact on their children’s participation in physical fitness, while the sub-healthy group shows no significant impact) are the factors influencing teenagers’ participation in physical fitness, while the mother’s education level significantly affects teenagers’ participation in physical fitness (*p* < 0.001). Research findings indicate that both the mother’s education level and the father’s health status are crucial factors influencing teenagers’ engagement in sports (18). Specifically, mothers with higher education levels have a deeper understanding of fitness and health, which positively influences their children’s participation in physical activities. This effect is particularly pronounced among older adolescents, where maternal education is positively correlated with their involvement in sports activities. Conversely, fathers’ health status also plays a crucial role; healthier fathers are more likely to engage in physical activities with their children, providing motivational models and constructive feedback that enhance children’s confidence and acceptance of health-oriented values (19).

The mechanisms through which family human capital influences adolescents’ sports participation are multifaceted. Highly educated mothers are more likely to recognize the importance of physical fitness and lifelong sports habits, thereby providing positive experiences and support to their children. They also serve as role models, actively participating in sports themselves and encouraging their children to engage in organized sports activities (20). On the other hand, fathers’ health status affects their ability to participate in physical activities with their children, thereby influencing their children’s attitudes and behaviors toward sports. Healthier fathers are more likely to engage in co-participation, offering constructive feedback and serving as motivational models (21). Highly educated mothers may influence their children through two primary pathways. First, they transmit health literacy via structured discussions on exercise physiology and injury prevention, facilitating the transfer of health knowledge. This enables adolescents to internalize the benefits of exercise and develop capabilities for risk assessment. Second, they adopt authoritative parenting styles that strategically encourage athletic engagement, activating observational learning through parental sports participation.

Family support, a critical component of family human capital, has been proven to be a positive predictor of children’s and adolescents’ participation in sports activities (22). Parents’ encouragement, support, role modeling, and attitudes positively correlate with their children’s engagement in physical activities (23). This support can take various forms, including providing resources, encouraging participation, and creating a supportive environment that fosters a healthy lifestyle. Additionally, family socio-economic status and income, often linked to parents’ education levels, influence the accessibility and affordability of sports for children, further impacting their participation in organized sports activities.

Future research should prioritize enhancing parental awareness of the relationship between health and physical activity through strategic interventions. These interventions could include family environmental modifications and structured parental engagement programs aimed at optimizing adolescents’ sports participation efficacy. By improving parental understanding and involvement, these programs can create a more supportive family environment that encourages healthy lifestyle choices and regular physical activity among adolescents.

### 4.2 The influence of the family’s natural capital endowment

The present study considered that the influence of family natural capital was not statistically significant on participation in fitness behavior (*p* > 0.05), but it had a significant impact on the degree of sports participation, and the family activity space was proportional to the frequency of youth sports participation.

Family natural capital refers to the natural means of production owned by the family that can promote teenagers’ sports fitness behavior. Under the influence of urbanization, the natural capital owned by families is limited. This study only includes the area owned by the family that can be used for fitness. The analysis shows that natural capital does not have a significant impact on participation in physical fitness activities. It may be because the motivation for participation is a result of multiple factors, including a positive family environment, parents’ attitudes toward sports fitness, role models, a positive family fitness atmosphere, the number of siblings, and interactive participation (24). Merely having a large family space or a wide yard does not constitute a motivation for sports participation, but it will affect the level of participation among teenagers who engage in sports fitness. For the youth group with sports fitness behavior and awareness, communities where high-income families reside may be more likely to offer sports and fitness facilities and equipment. The socioecological framework categorizes environmental influences on human development into four hierarchically organized systems: microsystem (immediate settings like family and school), mesosystem (interconnections between microsystems), exosystem (external environments indirectly affecting development), and macrosystem (overarching cultural values). Within this theoretical construct-as articulated in Bronfenbrenner’s ecological systems theory—the microsystem assumes particular salience during adolescence, encompassing proximal interactions within familial, scholastic, and community contexts. Our findings align with Bronfenbrenner’s proposition that physical environments operate as enablers of health behaviors but do not intrinsically motivate-health behaviors (25). Specifically, Household activity spaces (e.g., yards, home gyms) facilitate higher exercise frequency among already motivated adolescents, yet fail to overcome psychosocial barriers (e.g., screen time addiction, academic pressures) that suppress initial engagement (26).

In addition, the size of community activity venues and spaces has a positive impact on youth physical fitness activities, enhancing the accessibility of their participation. Although they are public places, this study did not include them. However, the quality of the community is related to the socioeconomic status of the family. Although not owned solely by the family, it can also be included as part of family resources. Informal sports will increase the participation rate of youth activities. Community activity venues reduce the distance between homes and sports facilities, most of which are free and do not require special or expensive equipment. However, participation in informal sports activities may be influenced by socioeconomic status. The likelihood of children and adolescents participating in informal sports activities is related to their surrounding environment, including family, community, and safety levels. Children and adolescents from low-income communities face restrictions in accessing sports and leisure facilities (27). Firstly, communities with a high level of safety are positively correlated with spontaneous active play, the rules for active play in the community are more stringent, and a good community environment provides safety guarantees for active participation (28). Secondly, the availability of community entertainment facilities is positively correlated with the participation of children and adolescents in sports, which has a positive impact on their sports participation (29). The affordability and accessibility of sports facilities in low-income communities are limited, while communities with higher socioeconomic status typically provide more sports facilities.

### 4.3 The influence of family economic capital endowment

There is no significant difference in the impact of family debt levels on the physical fitness activities of adolescents. This finding is likely due to the fact that current family mortgages have become a normative aspect of financial life. Most families do not curtail their investment and support for teenagers’ sports education because of mortgage debt. This result aligns with recent socioeconomic analyses suggesting that the type of debt—rather than the absolute magnitude of debt—moderates the impact on health behaviors (30). Mortgage obligations are culturally perceived as long-term investments in familial stability rather than as restrictive burdens, thereby minimizing their inhibitory effects on discretionary health expenditures. Higher-income households tend to maintain stable investments in sports activities, whereas low-income families are more likely to reduce fitness spending at lower debt thresholds. Despite varying debt levels, parental prioritization of children’s athletic development remains consistent. But the level of family income does have a statistically significant impact on the level of participation in physical fitness activities among adolescents, especially those organized fitness activities that require a certain level of financial support, which are directly linked to family economic capital. The level of family income represents a certain family socio-economic status. Research shows that family socio-economic status represents the level of available resources and family sports lifestyle, which is an important factor causing adolescent health inequality. The impact of economic status on health is closely related to behavior choice, and adolescent sports and fitness expenditure varies according to family income and socio-economic status (31).

There is a significant and direct correlation between the socio-economic status of parents and their children’s participation in sports (32). Children with higher socio-economic status can participate in sports more actively, while economic barriers restrict the participation of adolescents from low-income families in sports activities. Children and adolescents find it more challenging to participate in organized sports activities, potentially leading to a severe lack of physical activity among adolescents (33). Lower socio-economic status is often associated with poorer health behaviors, including overweight, reduced physical activity, and sedentary behavior. Currently, numerous studies indicate that children from lower socio-economic status groups exhibit declining physical health levels and face a higher risk of obesity compared to those from high-income families (34).

A recent study suggests that there are class differences in adolescents’ sports participation. Higher social classes are associated with greater involvement in organized sports activities, meaning that parents with higher incomes support their adolescents’ participation in both self-organized and organized sports activities (35). There is a strong correlation between socio-economic status and the physical development of children and adolescents, and those supported by their families are more active in participating in sports. On the one hand, investments by parents with higher socio-economic status enable teenagers to access better equipment and resources. On the other hand, the focus is on differences in fitness values, beliefs, and attitudes (36). The importance of health and awareness of health literacy are generally recognized among higher social classes. There is a positive correlation between parents’ socio-economic status and their involvement. Parents with high socio-economic status are more likely to support their children’s sports, such as encouraging them to become club members and setting an example through their own participation. Due to this group’s high level of sports participation, they usually have more time to commit, such as accompanying their children to participate in and watch sports events, providing transportation, etc. Families with higher economic resources are more likely to afford professional and organized sports courses, which will better improve their sports coordination ability (37). A three-year longitudinal study found that children with higher economic status had better bone development and physical coordination due to their participation in sports activities. On the contrary, teenagers from families with low economic status may face many obstacles restricting their participation in sports. First, parents may unintentionally provide incorrect health beliefs and unfavorable subjective norms. Second, due to work reasons, parents spend less time with and guiding their children to participate in sports activities, and usually only have limited time to acquire knowledge about physical health and support their children’s involvement in sports activities, making it more difficult for children to actively participate in physical exercise, leading to higher screen use time for this group (38).

Family is an important factor in the socialization of children, providing opportunities to acquire basic skills, habits, and knowledge. Parents mainly influence children through social skills and social cognitive ability, which plays a decisive role in the development of children’s social communication skills. Positive peer relationship experiences are very critical and important for their social psychological development and adaptation. Some studies have shown that family economic capital indirectly affects teenagers’ sports fitness behavior through peer support. As they age, they gradually reduce their dependence on their parents and learn the behavioral norms of peers. Peer support is positively correlated with teenagers’ sports participation (39). Support from parents and friends can promote children’s and teenagers’ regular participation in sports activities and help them develop and maintain a positive lifestyle (40). Children with good athletic abilities are more likely to have better peer relationships and acceptance, and lack of support from friends or parents is considered to be a barrier for children and adolescents to participate in physical fitness (41).

### 4.4 The influence of social capital endowment

The change in teenagers’ physical activity and sustained development behaviors is a complex social issue. Social networks are not merely static structures of relationships, but further research is needed on the most relevant factors, theories, and mechanisms at the level of social networks. Sports serve as a venue for fostering autonomy and long-term socialization (42). Within the context of family human capital, the number of family friends and relatives, as well as the quality of their relationship networks, have a statistically significant impact on teenagers’ physical fitness behaviors. Parents’ socioeconomic status is the most significant factor affecting children’s participation in sports, while intrafamilial social capital is the decisive factor. In cases of limited intrafamilial social capital, the quantity and quality of family friends and relatives become important factors influencing family sports and fitness concepts and participation, thereby promoting adolescents’ sports participation.

Besides parental support, this study incorporates whether the social support received by the family meets the demands for physical fitness as a key consideration. For instance, siblings within the family, contact with extended family, and relationships with other relatives and friends are all factors influencing adolescents’ participation in physical fitness. Social support from other family members or relatives can provide additional opportunities for sports participation and social development potential, thereby promoting adolescents’ sports participation. Moreover, the sense of efficacy influenced by peer effects not only affects adolescents’ choice of behavior but also determines the level of effort and ability to overcome obstacles during sports participation (5). The numerous social networking opportunities offered by high-quality social relationships have led to changes in parents’ awareness, goals, and knowledge regarding sports. Research shows that within the cognitive category, parents’ awareness and knowledge will affect the formulation of children’s goals, and positive social interactions promote physical activity among children and adolescents. Therefore, adolescents who receive high levels of support from both parents and peers are more likely to meet the recommended moderate to high-intensity physical activity levels (43). Parents’ behavioral and emotional investments influence the level of teenagers’ participation in sports. Sports serve as a bridge in social networks, facilitating emotional connections and experiences among peers through cumulative investments, and urging parents to obtain stronger emotions through their children’s sports experience. Because parents’ understanding and experience of their children’s sports environment are influenced by factors like education, income, cultural expectations, and family structure, there is a need to enhance the understanding of the theory of parental sports socialization.

Our findings on maternal education and paternal health align with global patterns while revealing culturally specific mechanisms in China’s collectivist context. Multigenerational households amplify maternal education effects through grandmothers’ childcare support-a phenomenon absent in Western nuclear families. The national college entrance exam (Gaokao) system concentrates academic workloads in junior high, explaining maternal education’s plateaued marginal benefits post-high school. Relationship networks (guanxi) further shape sports socialization: families with ≥10 local relatives report higher sports participation through resource-sharing kin density effects, while collective parenting norms reduce experimental sports exploration versus individualistic cultures. Two key limitations warrant attention: regional Confucian overrepresentation in Shandong data necessitates replication in southern hubs (e.g., Guangdong), and the recent “Double Reduction” policy’s temporal effects differ from established Western policy landscapes.

## 5 Strategy

### 5.1 Establish a social support system for family sports

Family education urgently requires social assistance. Social support for family education refers to the assistance relationship and its development process that is formed between individuals or organizations within the family and social environments in response to family education challenges or needs. This manifests as various forms of external assistance that families can receive. A social support system enables families and society to establish connections and receive beneficial support. In the systematic project of family physical education, school physical education serves as the foundation and key, guiding and deepening the process. Family physical education serves as a support and supplement to school education, while society extends the reach of school and family physical education, broadening the avenues for children and teenagers’ physical fitness and offering extensive sports resources and environments. Therefore, it is imperative to cultivate a ‘big family education perspective’ through complementary strengths and integration with schools and society, thereby fostering a concerted effort toward educating individuals with a shared vision and purpose, and collectively enhancing the physical health and well-being of adolescents. This necessitates the government’s leading role, actively mobilizing the social education ecosystem, activating and awakening a variety of high-quality social sports resources to serve the fitness of adolescents, and establishing a three-dimensional and diversified family sports social support system through close collaboration among families, schools, and society, ultimately aiming to enhance the quality of health education for adolescents.

### 5.2 Improve family physical education behavior

In family education, parents’ educational behaviors seriously lag behind their educational beliefs. Due to the weakening of the internal support network within the family and the lack of an established external support system, there is an urgent need for support and assistance from external sources. However, enhancing family physical education practices is a systematic project that requires the joint efforts of the state, schools, and society. Firstly, the government should play a role in legislative protection and overall supervision. While promoting parents’ educational beliefs, it should work together to provide specific services for family sports and explore ways to improve family physical education practices. Secondly, emphasis should be placed on family Confucianism, as the comprehensive quality of parents is a key factor in determining the quality of family education. “Teaching is what the elders impart and the younger ones follow; raising involves nurturing children to do good” (Eastern Han Dynasty, Xu Shen), which explains the role of parents as role models and demonstrators. Children carry the values and behaviors of their family as they enter school and society. It can be seen that the role of parents as role models can enable teenagers to be influenced by good ideas, behaviors, and habits within the family environment, while shaping good fitness awareness and value recognition, thereby gaining inherent advantages in the educational ecosystem. Therefore, through parents’ personal demonstration, they can showcase their values and behaviors to their children, promoting their understanding and experience, which is an important means to enhance family physical education practices. The family is the main place for the formation of teenagers’ sports fitness beliefs and behaviors.

### 5.3 Develop family sports promotion strategies

Given that family resources have been identified as a key factor in supporting and determining teenagers’ physical fitness activities, it is necessary to provide space for establishing family-based sports development strategies, position the family as a strong advocate for teenagers’ physical fitness activities, enhance and sustain the level of physical activity among family members, and integrate family sports activities into the school and community environments from a policy perspective. Firstly, it is essential to develop the theory of family sports education. The absence of a comprehensive theory in the modernization of education hinders the high-quality development of family sports. To improve the quality of family sports, it is necessary to innovate family education and sports theory, criticize and establish a theoretical framework for modern family education. Secondly, establish a family sports service guidance system. This system aims to enhance the ability and quality of family sports education and promote healthy physical activities among children and adolescents. It provides effective family sports guidance services for families. Therefore, it is necessary to emphasize the government’s leading role as the primary provider, enhance the participation awareness of schools, leverage schools as a link in diversified collaboration, and improve community engagement, balance community collaborative functions, and actively provide diverse information and resource support.

## 6 Conclusion

1. Family endowment has a positive impact on teenagers’ sports fitness behavior. The human capital, natural capital, economic capital, and social capital of families have a potential impact on the quantity and quality of teenagers’ sports participation. Under the normalization of epidemic prevention and control, family education has an impact that school education cannot.
2. Family-based interventions play an important role in the development of teenagers. Because parents play an important role in their children’s sports participation, it is necessary to build a social support system centered on family support on the basis of establishing a positive family sports environment.
3. To address the social disparities that exacerbate health disparities, special attention should be paid to adolescents with low parental education levels. It is crucial to strengthen family education on physical fitness awareness and behavior, which will positively impact their lifelong health and encourage the transformation of family education into active participation in teenagers’ physical fitness activities.

## Data Availability

The original contributions presented in the study are included in the article/supplementary material, further inquiries can be directed to the corresponding authors.
